# High enthusiasm about long lasting mentoring relationships and older mentors

**DOI:** 10.1186/s12909-019-1791-8

**Published:** 2019-09-23

**Authors:** Heba A. Mohtady, Karen D. Könings, Mohamed M. Al-Eraky, Arno M. M. Muijtjens, Jeroen J. G. van Merriënboer

**Affiliations:** 1 Medical Education Department, Fakeeh College for Medical Sciences, P.O 2537, Jeddah, 21461 Kingdom of Saudi Arabia; 2 Maastricht University, Maastricht, the Netherlands; 3 Microbiology &Immunology Department, Faculty of Medicine, Zagazig University, Zagazig, Egypt; 4 Imam Abdulrahman Bin Faisal University, Dammam, Saudi Arabia

## Abstract

**Background:**

Mentoring plays a pivotal role in workplace-based learning, especially in the medical realm. Organising a formal mentoring programme can be labor and time intensive and generally impractical in resource constrained medical schools with limited numbers of mentors. Hence, informal mentoring offers a valuable alternative, but will be more likely to be effective when mentors and protégés share similar views. It is therefore important to gain more insight into factors influencing perceptions of informal mentoring. This study aims to explore mentors and protégés’ perceptions of informal mentoring and how these vary (or not) with gender, age and the duration of the relationship.

**Method:**

We administered an Informal Mentor Role Instrument (IMRI) to medical practitioners and academics from Egypt, Pakistan and Saudi Arabia. The questionnaire was developed for the study from other validated instruments. It contained 39 items grouped into 7 domains: *acceptance*, *counselling*, *friendship*, *parenting*, *psychological support*, *role modelling* and *sociability*.

**Results:**

A total of 103 mentors and 91 protégés completed the IMRI. Mentors had a better appreciation for the interpersonal aspects of informal mentoring than protégés, especially regarding acceptance, counselling and friendship. Moreover, being older and engaged in a longer mentoring relationship contributed to more positive perceptions of interpersonal aspects of mentoring, regardless of one’s role (mentor or protégé).

**Conclusion:**

It can be concluded that the expectations of mentors and protégés differed regarding the content and aim of the interpersonal characteristics of their mentoring relationship. We recommend mentors and protégés to more explicitly exchange their expectations of the informal mentoring relationship, as typically practiced in formal mentoring. Additionally, in our study, seniority and lasting relationships seem crucial for good informal mentoring. It appears beneficial to foster lasting informal mentoring relationships and to give more guidance to younger mentors.

**Supplementary information:**

**Supplementary information** 10.1186/s12909-019-1791-8.

## Background

Mentoring is a relationship between a mentor (usually senior) and protégé (usually junior) that aims to guide personal and professional development over time [1–4]. Numerous benefits have been attributed to mentoring in undergraduate and postgraduate medical training as well as continuing professional development [5–7]. For instance, protégés learn to build their capacities, achieve learning outcomes, and hone their clinical [8, 9] and research skills [10, 11]. Mentored clinical faculty members were more satisfied with their department and institution than their non-mentored peers [6]. Mentors also feel greater productivity, career satisfaction, self-growth as leaders, pride, the ability to cope with conflicts, development of leadership skills and personal gratification [12]. According to the social exchange theory [13], both mentors and protégées interpret a negotiated trade of benefits, where mentors offer their connections, experience and support in return for personal affiliation, innovative ideas and appreciation by their protégés. Mentoring relationships foster emotional, cognitive and even spiritual transformation, provided that mentors and protégés have shared assumptions and ideas [14].

Mentoring can be either formal (i.e. institution-instigated) or informal (i.e. occurring spontaneously). Formal mentoring is essentially a task or an assignment and is typically perceived as such, whereas the informal relationship is a *choice*. The two types of mentoring are complimentary, not mutually exclusive. Informal mentoring provides a valuable alternative due to the spontaneous nature of the relationship [15]. Yet the absence of clear guidelines or a formal assignment may create uncertainty about mentors and protégés’ roles and expectations and about who should take the lead [16]. In addition, restriction of time and resources for both students and teaching staff has hindered the implementation of formal mentoring programs, particularly in novel medical schools [17] Moreover, limitations of effective formal mentoring programs were based on gender roles, cultural background, lack of opportunities of formal mentoring, disorganization and insufficient funding for human resources [18].

Conflicts may occur when a mentor assumes competing roles as a guide and an evaluator for the same cohort of protégés or when protégés betray trust, damage the mentor’s reputation, rely heavily on their mentor, ignore the mentor or are ungrateful to them. Unsuccessful informal mentoring may be attributed to poor implementation and discrepancies in protégé-mentor perceptions. That said, it is important to learn more about the factors that influence perceptions of informal mentoring [15, 19, 20].

Research on mentoring to date has been directed in large part towards formal mentoring that is embedded within a formal curriculum. Largely unexplored, however, are the factors that enhance initiation, perception and preservation of these informal relationships by physicians or faculty [21]. Scant attention has been paid to the dynamics of informal mentoring, potential differences in perceptions between mentors and protégés and the influence of gender, age and the duration of the relationship on the mentoring experience. The present research will seek to address this latter deficiency.

### Perceptions of informal mentoring

Informal mentoring relationships are fluid in nature and rest on implicit and diverse expectations between mentor and protégé. Sometimes one of the partners does not even acknowledge being in a mentoring relationship. In order for mentors and protégés to benefit the most from the relationship, awareness of their distinct, yet complementary roles, is key.

Perceptions of informal mentoring in the medical realm may be mediated by several factors that operate at the *personal*, *interpersonal* and *professional* level [7]. At the personal level, age, gender, race and cultural background of mentors and protégés may moderate their experiences [19, 22]. At the interpersonal level, the quality of psychological and emotional support received by the protégés may affect the relationship in different forms [2, 5]. As role models, informal mentors convey their valuable tacit knowledge and insights about clinical practice, professionalism, ethics and the art of medicine. Finally, at the professional level, perceptions are contingent upon the ability of informal mentors to offer careers guidance and protection and act as their protégé’s coach and sponsor [16, 23, 24].

### Age as a variable

Although mentors have traditionally paired with protégés who were 8–15 years younger of age, there is a growing tendency towards similar-age (peer-to-peer) or even reverse-age mentoring. We postulate that age can impact the mentoring experience because junior and senior protégés have different perceptions of what is the *ideal mentor* and varying expectations from the relationship [25]. Also, older protégés need more psychological support and career mentoring. We therefore set out to examine the effect of age on different aspects of mentoring support.

### Gender as a variable

Gender may also moderate the mentoring experience. Expectations and behaviours of mentors and protégés have been shown to differ between sexes. Males, for instance, were more likely to serve as mentors and mainly provided career-related advice [26], while their female peers focused more on the provision of psychological support to their protégés of both genders in their nascent relationship [27]. A multi-institutional study reported that female protégés received more psychosocial support from female mentors than did their male counterparts [28]. The aforementioned differences may have a bearing on the way mentors and protégés perceive the informal mentoring relationship.

### Duration of the relationship

The duration of the informal mentoring relationship may impact on mentoring experiences. It can vary from a few months up to a lifetime. An example of a short-lived mentoring experience was reported by Cellini and colleagues who organised a *speed mentoring* programme in which protégés each met with six mentors for ten minutes per pairing [29]. Three months later, mentors sent protégés reminders on the agreed action plan via email. Despite the ultra-short period of contact, participants felt it offered them opportunities to network and almost 60% of the mentoring dyads were motivated to meet again after the programme.

The continuation of a relationship depends on open communication, availability, mutual respect, clear goal setting, role modelling, the mentor’s coaching skills, and the presence of a protégé portfolio [3, 30, 31]. Whether and how the duration affects mentors and protégés’ perceptions is one of the questions that we seek to address in the current study.

To recap, the present study investigates the dynamics of informal mentoring by measuring the differences in perceptions between mentors and protégés and how these perceptions vary (or not) with *age*, *gender* and the *duration* of the relationship. To this end, the following two research questions will be addressed: (1) Do mentors and protégés differ in their perceptions of the interpersonal aspects of informal mentoring? (2) How do mentors and protégés’ perceptions of informal mentoring vary with their age, gender and the duration of the relationship?

## Methods

### Participants

We targeted medical practitioners, academic staff members and graduate and doctoral students in governmental institutions in Egypt, Pakistan and Saudi Arabia. We asked voluntary participants if they were presently or recently engaged in an informal mentoring relationship and whether they fulfilled the role of mentor or protégé in this relationship.

### Materials

We searched the literature for instruments that measure perceptions of mentoring relationships [1, 32–37]. Based on the items of these instruments on formal mentoring, we developed a 39-item questionnaire which we coined the ‘Informal Mentor Role Instrument’ (IMRI) and that could be administered to both mentors and protégés. All items were taken from other internationally published valid questionnaires and the number of items was based on the original subscales. These items were grouped into seven domains about interpersonal aspects of informal mentoring, specifically: acceptance (6 items), counselling (9 items), friendship (5 items), parenting (3 items), psychological support (3 items), role modelling (10 items), and sociability (3 items). Table 1 lists a few item samples for each domain and the reference source from which they were drawn.

**Table 1:** Item samples for each of the seven subscales of the Informal Mentor Role Instrument (IMRI)

Subscale	Number of items	Sample items	Reference
Acceptance/confirmation	6	● The mentor accepts the protégé as a competent professional.	[37]
		● The mentor conveys feelings of respect for the protégé as an individual.	[36]
Counselling	9	● The mentor demonstrates good listening skills with the protégé’s conversations.	[36]
		● The mentor encourages the protégé to talk openly about anxiety and fears that detract from his/her work.	[36]
		● The mentor serves as a sounding board for the protégé to develop and understand him/herself.	[37]
Friendship	5	● The mentor invites the protégé to join him/her for lunch.	[36]
		● The mentor is someone the protégé can trust.	[37]
Parenting	3	● The mentor reminds the protégé of one of his/her parents.	[37]
Psychosocial support	3	● The protégé shares personal problems with the mentor.	[35]
Role modelling	10	● The protégé tries to imitate the work behaviour of his/her mentor.	[36]
		● The mentor serves as a role model for the protégé.	[37]
		● The mentor represents who the protégé wants to be.	[37]
Sociability	3	● The mentor and the protégé frequently have one-to-one, informal social interactions outside the work setting.	[37]

All items were rated on a 5-point Likert scale, ranging from 1 (strongly disagree) to 5 (strongly agree), the higher scores representing more favourable perceptions. We assessed the reliability of the IMRI by calculating Cronbach’s alpha for each of the seven subscales. The alpha per subscale ranged between .90 and .98 for protégés and between .51 and .89 for mentors (see Table 2). The subscale ‘psychosocial support’ had a good alpha for protégés (.90) and an insufficient alpha for mentors (.51). We decided to use the subscale in the analyses but interpret the results with caution. For other subscales, all Cronbach’s alphas were acceptable, as for the newly developed scales, a Cronbach’s alpha > 0.6 has been considered acceptable.

**Table 2:** Descriptives of the seven subscale scores and demographics (*n* = 194)

IMRI subscale	Number of items	Mean^a^	SD	Reliability (Cronbach’s alpha)		
				Protégé^b^	Mentor^b^	
Acceptance	6	3.71	0.84	0.95	0.65	
Counselling	9	3.76	0.87	0.98	0.81	
Friendship	5	3.43	0.90	0.92	0.70	
Parenting	3	3.40	0.99	0.94	0.80	
Psychosocial support	3	3.63	0.93	0.90	0.51	
Role Modelling	10	3.70	0.80	0.97	0.79	
Sociability	3	2.92	0.99	0.91	0.89	
Variable		Scale	Min.	Max.	Mean	SD
Age		Years	24	63	37.9	10.8
Duration		Months	1	240	20.5	24.8
LgDur		+ 1 = duration × 10	0	2.38	1.11	0.44
Variable		Scale			Numbers	Percentages
Gender		0: Male, 1: Female			92 102	47.4, 52.6%
Role^b^		0: Protégé 1: Mentor			91 103	46.9, 53.1%

^a^ Higher scores representing more favourable perceptions

^b^ Participant’s role in the current or most recent mentoring relationship (variable role)

Apart from the items on the aforementioned domains, the IMRI asked participants to indicate their role (mentor or protégé), age (in years), gender (male or female), and the duration of the current or most recent informal mentoring relationship (in months). To make the IMRI applicable to both mentors and protégés, we changed the original wording of the items by eliminating all mentions of ‘my mentor’, ‘my protégé’, ‘me’ or ‘you’ and replacing them with ‘the protégé’ or ‘the mentor’, as appropriate. Other adjustments were the replacement of all references to ‘school’ and ‘education’ by ‘work in the medical education field’ and the rephrasing of items to be written in the positive. We produced both an English and an Arabic paper version of the questionnaire to make it intelligible to all participants. The English questionnaire was also available in online format.

### Procedure

We administered the Arabic paper version in person to attendees of departmental meetings, workshops and seminars at the medical college of Zagazig University, Egypt, and collected their responses on the spot. The English version was distributed both online and in paper format to graduate students and faculty members at the medical college of Riphah University in Pakistan and to faculty members at the college of medicine, University of Dammam, Saudi Arabia. We informed participants of the study’s purpose verbally while distributing the paper version or, in the case of the online version, through a short standard paragraph posted at the beginning of the survey.

After having explained the rationale of the research, the volunteered participants were asked to sign an electronic consent form. Then the survey started with the items that asked for participants’ background information. Participants were consequently asked if they had any past experience with informal mentoring, which was defined as follows: ‘Informal mentoring is based on natural personal matching and mutual interests between a junior and a senior person. Its ultimate outcome is professional development and considerable impact and satisfaction for both.’ Participants were instructed to enter ‘0’ and to stop completing the questionnaire if they had never had an informal mentoring experience. Ethical clearance was obtained to administer the IMRI English & Arabic versions of the used questionnaire (Additional file 1).

### Data analysis

To be able to answer the first research question, we computed the mean perception scores per domain for each group (mentors and protégés) and compared these average group scores. We addressed the second research question by investigating the relation of participants’ age and the duration of the current or most recent informal mentorship with the domain scores, which yielded the continuous variables of Age (in years) and Duration (in months). Since we expected the distribution of Duration to be considerably skewed, we also used the log10-transformed version of Duration in the analysis, which we labelled ‘LgDur’. We considered participants’ gender as a potential confounder, and therefore included Gender (0: male; 1: female) in the analysis as a control variable. Moreover, as the relationship between a subscale and Age and Duration could vary in accordance with participants’ role of mentor or protégé, we included the variable ‘Role’ in the analysis as a grouping variable (0: protégé; 1: mentor). In a final step, we obtained descriptive statistics of the above-mentioned variables and investigated the factors explaining the variation in each domain score in a multiple regression analysis, using the domain score as dependent variable and Age, LgDur, Role, Gender and the interactions RoleLgDur (= Role×LgDur) and RoleAge (= Role×Age) as independent variables. For ease of interpretation and to improve numerical stability in the regression analysis, we centred the continuous variables of Age and LgDur. That is, we subtracted the variable’s average value from the original value, as in Age centred = Age – average (Age). In the stepwise regression analysis, we first entered Age, LgDur, Role and Gender into the model. After that, we used a backward procedure to investigate the interactions which we retained only if found to contribute significantly to the explanation of the variance in the dependent variable. We used the standard regression coefficient (src) as an indicator of effect size, applying Cohen’s rule of thumb that values of 0.1, 0.3 and 0.5 indicate a small, moderate, and large effect, respectively. Results were considered statistically significant when *p* < 0.05. We used IBM SPSS Statistics, version 24, for all statistical analyses.

## Results

A total of 226 participants completed the questionnaire, 180 of which were from Egypt, 32 from Pakistan and 14 from Saudi Arabia. From these participants, 25 were excluded because they failed to indicate which role they fulfilled (protégé or mentor) in their most recent mentoring relationship. Another six participants were excluded because they did not answer 7 or more of the 39 items in total, leaving 194 participants whose data formed the input to our analysis. Following the guideline that you need minimum 50 + 8 x ‘the number of independent variables’ participants per variable in your statistical model, our sample size of 194 respondents adequate to test statistical models with four independent variables.

As anticipated, the distribution of the variable Duration demonstrated unacceptably high values of skewness and kurtosis (4.90, 36.21), while the logarithmically transformed version of Duration, LgDur, exhibited considerably better values (− 0.44, 0.70) that were within the acceptable range. Tables 2 and 3 provide an overview of the results, with Table 2 presenting descriptives and reliabilities (Cronbach’s alpha) of the scores on the seven subscales of the IMRI, as well as descriptives for the demographic variables Age, Duration, LgDur, Gender and Role. Table 3 displays the data explaining the relation between each subscale score and Age, Duration, Gender, Role and the interactions Role Age and RoleLgDur, as they resulted from the multiple regression analysis. We did not report the data pertaining to the independent variables that did not exhibit any significant contribution, for which reason we did not include the non-significant interactions ‘Role Age’ and ‘RoleLgDur’ either. Regression coefficient *b* of the intercept represents the domain score of a male protégé (Gender = 0; Role = 0) of average age 1(37.9 years) whose current or most recent mentoring relationship lasted 12.9 months which corresponds to the average LgDur (1.11) or 10^1.11^.

**Table 3:** Data from the Multiple Regression Analysis detailing the effect of role, gender, age and duration of the relationship on the perceptions of participants (*n* = 194)

Dependent variables (IMRI subscales)	Independent variables of informal mentoring relationship													
	Intercept		Role 0 Protégé (n. 91) 1 Mentor (n. 103)			Gender 0 Male (n. 92) 1 Female (n. 102)			Age (in years)			LgDur (+ 1 means duration × 10)		
	*b*	*p*	*b*	*sc*	*p*	*b*	*sc*	*p*	*b*	*sc*	*p*	*b*	*sc*	*p*
Acceptance	3.42	.001	.399	.24	.012							.355	.18	.008
Counselling	3.45	.001	.393	.23	.011				.020	.25	.007	.438	.22	.001
Friendship	3.03	.001	.489	.27	.003	.262	.15	.028				.515	.25	.001
Parenting	3.54	.001							.027	.29	.003	.349	.15	.036
Psychosocial support	3.61	.001										.368	.17	.019
Role Modelling	3.54	.001										.506	.28	.001
Social	2.64	.001										.343	.15	.039

Note: The table only presents significant effects; the interactions ‘RoleAge’ and ‘RoleLgDur’ resulted non-significant, and were therefore omitted

*b*: regression coefficient

*sc*: standard regression coefficient (not defined for the Intercept)

*p*: two-sided *p* value of the *t*-test against *b* = 0

With respect to our first research question as to whether mentors and protégés have different perceptions of mentoring, we found that mentors on average had significantly higher perception scores for Counselling, Friendship and Acceptance (increases of 0.4 to 0.5) than protégés, the corresponding *sc*, which varied from .23 to .27, indicating effects of moderate size. Hence, compared to protégés, mentors had more positive perceptions of three out of seven domains. As to our second research question about the influence of age, gender and the duration of the mentoring relationship on perceptions, we found moderate effects (*sc* .25 and .29) of age for Counselling and Parenting, with perception scores increasing by 0.020 and 0.027, respectively, with each additional year. Furthermore, we found that perception scores for all subscales increased significantly as relationships lasted longer, the regression coefficients *b* showing increments of 0.34 to 0.52 each time Duration was multiplied by a factor of 10. Corresponding effect sizes were small to moderate, as evidenced by the *sc’s* which ranged from 0.15 to 0.28. Controlling for gender, we found it to be a confounder only in the domain of Friendship, since female participants on average had a 0.262 higher score for Friendship than their male peers, the *sc* of 0.15 signalling a small effect. Finally, the non-significance of the interactions ‘Role Age’ and ‘RoleLgDur’ confirmed that being older and engaged in a longer mentoring relationship contributed to more positive perceptions of interpersonal aspects of mentoring, regardless of one’s role (mentor or protégé).

## Discussion

The present study examined how participants’ role (mentors’ vs protégés), their age, gender and the duration of the relationship influenced perceptions of informal mentoring relationships. Among a heterogeneous cohort of informal mentors and protégés, we found significant differences in perceptions of mentorship domains based on roles. Further, we found that age might be contributing to such perceptions across both roles. In terms of roles, mentors had more positive perceptions in the domains of *acceptance*, *counselling* and *friendship*, which may be because mentors were only connected with the protégés they liked [38, 39]. This observation ties in perfectly with the assertion that interpersonal comfort is essential to the success of informal mentoring relationships.

As for age, there was a positive correlation between age and mentors and protégés’ perceptions in the domains of *counselling* and *parenting*. Younger mentors reported less favourable perceptions of informal mentoring, which can possibly be attributed to an exaggerated sense of their importance and narcissistic personality traits. At the start of their career, the less experienced mentors believed they had the right to unlimited success and may take advantage of others [40].

The notion that age is of significance to career choice, counselling and professional development has previously been reported by studies on mentoring in the medical context [1, 5, 23]. Strikingly, these observations did not apply only to the classical mentoring dyad in which mentors are senior to their protégés. Our study has demonstrated that the age of both mentor and protégé affects perceptions of mentoring. Peer-to-peer mentoring helps both mentors and protégés to learn communication, documentation and leadership skills [41, 42]. PhD students in particular, can benefit from peer mentoring as their colleagues can assist them and socialise them into their role as future scholars [43]. Mid-career faculty are more likely than late-career faculty to be interested in serving as mentors [44].

Mentorship could present itself in different patterns. Among these patterns, the hierarchical relationship between senior and junior members represents the commonly encountered model [45]. Cultural differences include differences in styles of learning, expression of thoughts, and perceptions of different forms of relationships. For instance; perception of hierarchy in academia differs as candidates might be disinclined to speak up out of culturally expected respect for the senior individuals, or to consider ‘listening’ as a more suitable attitude to learning [46, 47].

With respect to duration, our study advocated that longer relationships were associated with more positive perceptions of different aspects of informal mentoring, notably *friendship* and *role modelling*. This seems plausible, since mentoring dyads are more likely to become friends when the relationship lasts longer. The reverse may also be the case: when partners are united in friendship and protégés perceive their mentors as role models, the relationship may persist. A ‘*friend’* and a ‘*role model*’ can be the personal qualities partners see in the other in the early stage of identification, when mentors and protégés first connect with each other [38, 48].

Another determinant of mentoring perceptions, albeit to a minor extent, was gender. A meta-analysis was conducted by O’Brien et al., 2010 to address mentoring relationships from the perspective of gender differences between mentor- and protégé. According to this study, males described giving more advice on career development than female mentors. Equally, female mentors conveyed providing more psychosocial support than male mentors. There were no gender differences for protégés regarding career development, However, male protégés stated obtaining less psychosocial support than female protégés [49].

Women were more inclined to infuse a sense of *friendship* into the informal mentoring relationship. Other female-specific features emphasised in the literature are that female protégées expect their informal mentors to be their counsellors, coaches, teachers and in some occasions even their sponsors [50]. Moreover, women not only like to climb the ladder (as protégées), but also to hold it for others (as mentors) [51], which demonstrates their friendliness and readiness to play both roles. Unlike their male peers who are concerned with career-related advice, female mentors usually aspire to provide more psychological support [27], sometimes indirectly affecting career choices. In surgery, for instance, female protégées’ choice to become a surgeon was influenced by their female surgical mentors whom they perceived as role models [52]. Finally, although cross-gender mentoring relationships have been shown to be more resilient compared to same-gender ones [53], women generally prefer mentors who are of the same sex [48].

The above results should be interpreted in the context of two phenomena in interpersonal relationships that have been of great interest to social scientists since the 1950s: ‘*homophily’* and ‘*liking attraction*’ [54, 55] *.* Homophily or similarity attraction is the tendency of mentors and protégés to engage with those who share the same core values, attitudes and characteristics*.* Similarity breeds connection, a principle that governs relationships of all time, including marriage, friendship and work. Without a baseline understanding and similarity between mentors and protégés, mentoring relationships are likely to fail [28, 39]. The second form of attraction, *liking*, means that people are drawn to those who seem to like them [56]. Interestingly, both types of attraction were recently found highly interconnected and to contribute to *trust* between two individuals [57].

As was highlighted before, Social Exchange Theory [13] is often invoked to explain the underlying mechanisms of informal mentoring and associated developmental changes in both members. As such, informal mentoring is usually portrayed as a *pragmatic*, albeit sophisticated, transactional beneficial relationship that is based on *give* and *take*. By suggesting a couple of theoretical implications, this study made a humble attempt to contribute to this understanding and to offer more insight into the motivations that drive mentor-protégé dyads. The study recalls that mentoring relationships do not exist in a vacuum. We therefore must acknowledge and further examine their context to unearth the personal, interpersonal and circumstantial variables that can make or break these relationships.

However, some limitations need addressing. First, the general pattern of informal mentoring relationships that emerged was restricted by the variables we selected, as is the case in most quantitative studies. Moreover, we did not match mentors’ perceptions with those of their partner protégés, and we certainly invite future research to make up for this shortcoming. The large standard deviation of the mean scores on the IMRI subscales underlines the importance of future studies investigating mentor-protégé pairs, so they reflect on the same mentoring relationship. Furthermore, the IMRI was, although based on items from internationally published instruments, not fully validated in the countries of this study. A future validation study is recommended. Finally, we solely focused on perceptions of the informal mentoring relationship, without exploring whether favourable perceptions were also associated with better outcomes, such as a better receptivity to feedback by protégés [58]. Previous research has suggested that long-term mentoring relationships indeed contribute to the provision of more open and honest feedback [59] and better use of feedback for professional development (Sargeant et al., in press). We welcome future research into the question of whether or not learning and professional development are more effective when relationships last longer or in case of seniority in different cultures.

## Conclusion

Mentoring can be one of the most fulfilling and transformative relationships we experience at work, but effectiveness cannot be taken for granted. Mentors had a better appreciation for the interpersonal aspects of informal mentoring than protégés. Moreover, perceptions of both mentors and protégés improved with age and the duration of the mentoring relationship in our study. We therefore recommend nurturing lasting informal mentoring relationships and to give more guidance to younger mentors and protégés.

## Supplementary information

**Additional file 1:** English and arabic versions of the used questionnaire. (DOCX 31 kb)

## Data Availability

The datasets used and/or analysed during the current study are available from the corresponding author on reasonable request.

## Abbreviation

IMRI: Informal Mentor Role Instrument
