# Supplementary material for: High enthusiasm about long lasting mentoring relationships and older mentors
Source: BMC Med Educ. 2019 Sep 23;19:364. doi: 10.1186/s12909-019-1791-8 (PMC6757421; doi:10.1186/s12909-019-1791-8)
Supplement: Supplementary file 1 — Additional file 1: English and arabic versions of the used questionnaire. (DOCX 31 kb) [file 12909_2019_1791_MOESM1_ESM.docx]

| to | | **Please answer the following questions:** |  | | **Date:**  **Participant Code:** | | | |
| --- | --- | --- | --- | --- | --- | --- | --- | --- |
| 1 | | Have you had an experience with informal mentoring before?  Definition: “Informal mentoring is based on natural personal matching and mutual interests between a junior and a senior person. Its ultimate outcome is professional development and considerable impact and satisfaction for both.” | Yes/No | | | | | |
| 2 | | If yes, how many times have you been in involved in such informal relationship? |  |  |  |  |  |  |
| 3 | | If you currently are involved in an informal mentoring relationship, what is your role? | Mentor | Protégé | Both | |  |  |
| 4 | | If you currently fulfil both roles, which one is dominant for you?  Please fill out this questionnaire with this role in mind. | Mentor | Protégé |  |  |  |  |
| 5 | | What is your gender? | Male | Female |  |  |  |  |
| 6 | | What is your age? | Date Of Birth: |  | Age (years): | | |  |
| 7 | | What is your speciality? |  | | | | | |
| 8 | | What is your current Job Status? |  | | |  |  |  |
| 9 | | In your current informal mentorship relationship, are both parties the same gender? | Yes/No | | | | | |
| 1310 | What is the What is the duration of this current informal mentorship since its beginning till now (in months)? | | | | | | | |

This questionnaire was developed as part of a research project on informal mentorship within the medical field. The items were originally developed by multiple scholars (Berk et al., 2006; Castro & Scandura, 2004; Noe, 1988; Ragins & Mcfarlin, 1990). These items were further modified for relevance and applicability of our study by Prof. Heba Mohtady, Dr. K. D. Könings, Dr. Mohamed A Aleraky, and Prof. J.J.G. Merriënboer. Your participation in this study is highly appreciated.

The following items present different statements and questions about your experiences in your most recent informal mentorship relationship. Please choose the degree to which you agree with each of the statements.

|  | **For each item, please indicate how much you agree with the statement** | **1** | **2** | **3** | **4** | **5** |
| --- | --- | --- | --- | --- | --- | --- |
|  |  | **Strongly Disagree** | **Disagree** | **Neutral** | **Agree** | **Strongly Agree** |
| 1 | The mentor thinks highly of the protégé. |  |  |  |  |  |
| 2 | The mentor accepts the protégé as a competent professional. |  |  |  |  |  |
| 3 | The protégé tries to imitate the work behaviour of his/her mentor. |  |  |  |  |  |
| 4 | The mentor shares personal experiences as an alternative perspective to the protégé’s problems. |  |  |  |  |  |
| 5 | The mentor shared ideas with the protégé. |  |  |  |  |  |
| 6 | The protégé tries to model his/her behaviour after the mentor. |  |  |  |  |  |
| 7 | The mentor is someone the protégé can trust. |  |  |  |  |  |
| 8 | The mentor encourages the protégé to try new ways of behaving in his/her job. |  |  |  |  |  |
| 9 | The mentor is someone the protégé can confide in. |  |  |  |  |  |
| 10 | The mentor provides support and encouragement. |  |  |  |  |  |
| 11 | The mentor is someone the protégé identifies with. |  |  |  |  |  |
| 12 | The mentor invites the protégé to join him/her for lunch. |  |  |  |  |  |
| 13 | The protégé will try to be like the mentor when he/she reaches a similar position in his/her career. |  |  |  |  |  |
| 14 | The mentor serves as a role model for the protégé. |  |  |  |  |  |
| 15 | The protégé agrees with the mentor's attitudes and values regarding work in medical field. |  |  |  |  |  |
| 16 | The mentor treats the protégé like a son/daughter. |  |  |  |  |  |
| 17 | The mentor conveys empathy for the concerns and feelings the protégé discussed with him/her. |  |  |  |  |  |
| 18 | The mentor reminds the protégé of one of his/her parents. |  |  |  |  |  |
| 19 | The protégé respects his/her mentor’s ability to teach others. |  |  |  |  |  |
| 20 | The mentor and the protégé frequently socialize one-on-one outside the work setting. |  |  |  |  |  |
| 21 | The mentor asks the protégé for suggestions concerning problems she/he encounters at work. |  |  |  |  |  |
| 22 | The mentor is like a father/mother to the protégé. |  |  |  |  |  |
| 23 | The mentor encourages the protégé to talk openly about anxiety and fears that detract from his/her work. |  |  |  |  |  |
| 24 | The mentor discusses the protégé’s questions or concerns regarding feelings of competence, commitment to advancement, relationships with peers and supervisors or work/family conflicts. |  |  |  |  |  |
| 25 | The mentor represents who the protégée wants to be. |  |  |  |  |  |
| 26 | The protégé admires his/her mentor’s ability to motivate others. |  |  |  |  |  |
| 27 | The protégé exchanges confidences with the mentor. |  |  |  |  |  |
| 28 | The mentor conveys feelings of respect for the protégé as an individual. |  |  |  |  |  |
| 29 | The protégé shares personal problems with the mentor. |  |  |  |  |  |
| 30 | The mentor guides the protégé’s personal development. |  |  |  |  |  |
| 31 | The mentor and the protégé frequently have one-on-one, informal social interactions outside the work setting. |  |  |  |  |  |
| 32 | The mentor keeps feelings and doubts the protégé share with him/her in strict confidence. |  |  |  |  |  |
| 33 | The mentor interacts with the protégé socially outside of work. |  |  |  |  |  |
| 34 | The mentor demonstrates good listening skills with the protégé’s conversations. |  |  |  |  |  |
| 35 | The protégé considers the mentor to be a friend. |  |  |  |  |  |
| 36 | The mentor and the protégé frequently get together informally after work by ourselves. |  |  |  |  |  |
| 37 | The protégée respects and admires the mentor. |  |  |  |  |  |
| 38 | The mentor serves as a sounding board for the protégé to develop and understand him/herselves. |  |  |  |  |  |
| 39 | The mentor guides the protégé’s professional development. |  |  |  |  |  |
| **Would you like to add anything about your experiences that was not covered above?** | | | | | | |

**Arabic version of the questionnaire**

**تم إعداد هذا الاستبيان كجزء من مشروع البحث حول النصح الغير رسمي فى المجال الطبي. قام العديد من الدارسين بتطوير العناصر (بيرك وآخرون، 2006؛ كاسترو و سكاندورا، 2004؛ نوي، 1988؛ راجينس و مكفارلين، 1990). تم تعديل هذه العناصر لأهميتها وقابليتها للتطبيق لدراستنا عن طريق البروفيسور/ هبة مهتدي، والدكتور/ ك. د. كونينجس، والدكتور/ محمد العراقى, و البروفيسور / جي جي جيه ميرينوبير، نقدر مساهمتك في هذه الدراسة.**

|  | برجاء الإجابة على الأسئلة التالية |  |  | | التاريخ: |
| --- | --- | --- | --- | --- | --- |
| 1 | هل لديك خبرة في النصح الغير رسمي من قبل؟  التعريف: " النصح الغير رسمي هو علاقة تنشأ بين شخصين احدهما اعلى فى المكانة الوظيفية و والشخص الاخر أصغر؛ ترتكز على وجود تطابق و تناغم طبيعى بين الشخصيتين وتكون محصلتها النهائية ذات تأثير كبير عليهما وتؤدى الى الرضا و التنمية المهنية لكليهما | نعم / لا | | | |
| 2 | إذا كانت الإجابة نعم، كم عدد المرات التي شاركت فيها في النصح الغير رسمي |  | | | |
| 3 | إذا كنت حاليا مشترك في علاقة نصح غير رسمية، فما هو دورك؟ | ناصح | مشمول بالرعاية (من يتم نصحه) | | كلاهما |
| 4 | إذا كنت تقوم كلا الدورين، فإيهما يهيمن عليك أكثر؟  برجاء وضع هذا الدور بعين الاعتبار عند ملء هذا الاستبيان | ناصح | مشمول بالرعاية | |  |
| 5 | ما هو جنسك؟ | ذكر | أنثى | |  |
| 6 | كم عمرك؟ | تاريخ الميلاد | | العمر بالسنوات | |
| 7 | ما هو تخصصك؟ |  | | |  |
| 8 | ما هو الوضع الوظيفي الحالي؟ |  | | |  |
| 9 | في علاقة النصح غير الرسمية، هل كلا الطرفين نفس الجنس | نعم /لا | | | |
| 10 | ما هي فترة علاقة النصح غير الرسمية هذه ، منذ بدايتها حتى الأن (بالشهور)؟ |  | | | |

|  | لكل عنصر، برجاء الإشارة إلي مدى موافقتك على هذه البيانات | 1  غير موافق بشدة | 2  غير موافق | 3  محايد | 5  موافق بشدة |
| --- | --- | --- | --- | --- | --- |
| 1 | يقبل الناصح المشمول بالرعاية كشخص لديه القدرة على الاحتراف مهنيا |  |  |  |  |
| 2 | يحاول المشمول بالرعاية محاكاة سلوك العمل الخاصة بناصحه |  |  |  |  |
| 3 | يقوم الناصح بمشاركة الخبرات الشخصية مع المشمول بالرعاية كوجهة نظر بديلة لمشاكله |  |  |  |  |
| 4 | يقوم الناصح بمشاركة الأفكار مع المشمول بالرعاية |  |  |  |  |
| 5 | يحاول المشمول بالرعاية نمذجة سلوكه بعد المدرب |  |  |  |  |
| 6 | يهتم الناصح اهتماما شخصيا بمهنة المشمول بالرعاية |  |  |  |  |
| 7 | الناصح هو الشخص الذي يستطيع المشمول بالرعاية الثقة به( الوثوق به) |  |  |  |  |
| 8 | يقوم الناصح بتشجيع المشمول بالرعاية لتجربة وسائل جديدة للتعامل/التصرف أثناءالعمل |  |  |  |  |
| 9 | الناصح هو مأمن أسرار المشمول بالرعاية |  |  |  |  |
| 10 | يقدم الناصح الدعم والتشجيع |  |  |  |  |
| 11 | يساعد الناصح المشمول بالرعاية في تنسيق أهدافه المهنية |  |  |  |  |
| 12 | المشمول بالرعاية يتفق مع صفات شخصية كثيرة فى الناصح |  |  |  |  |
| 13 | يدعو الناصح المشمول بالرعاية لتناول الغذاء معه |  |  |  |  |
| 14 | سيحاول المشمول بالرعاية أن يكون مثل الناصح عند وصوله لوظيفة مماثلة في مساره الوظيفي |  |  |  |  |
| 15 | يتقدم الناصح باعتباره نموذجا يحتذى به للمشمول بالرعاية |  |  |  |  |
| 16 | يوافق المشمول بالرعاية على قيم وسلوك الناصح بخصوص العمل في المجال الطبي |  |  |  |  |
| 17 | يعامل الناصح المشمول بالرعاية كأبنه أو أبنته |  |  |  |  |
| 18 | يتعاطف الناصح مع المخاوف والمشاعر التي يشعر بها المشمول بالرعاية والتي يناقشها معه |  |  |  |  |
| 19 | يذكر الناصح المشمول بالرعاية بأحد من والديه |  |  |  |  |
| 20 | يحترم المشمول بالرعاية قدرة الناصح على تعليم الأخرين |  |  |  |  |
| 21 | في كثير من الأحيان يقوم الناصح والمشمول بالرعاية بالتواصل معا اجتماعيا خارج نطاق العمل |  |  |  |  |
| 22 | يسأل الناصح المشمول بالرعاية أي اقتراحات تخص المشكلات داخل نطاق العمل |  |  |  |  |
| 23 | الناصح مثل الأب أو الأم بالنسبة للمشمول بالرعاية |  |  |  |  |
| 24 | يشجع الناصح المشمول بالرعاية للتحدث بحرية عن مخاوفه وقلقه الذي يساوره في العمل |  |  |  |  |
| 25 | يناقش الناصح تساؤلات المشمول بالرعاية أو مخاوفه بخصوص الشعور بالكفاءة، الالتزام بالتقدم، العلاقات مع الأقران والمشرفين أو نزاعات الأسرة أو العمل |  |  |  |  |
| 26 | يمثل الناصح للمشمول بالرعاية من يرغب ان يكون |  |  |  |  |
| 27 | يعجب المشمول بالرعاية بقدرة الناصح على تحفيز الأخرين |  |  |  |  |
| 28 | يتبادل المشمول بالرعاية الثقة مع الناصح |  |  |  |  |
| 29 | ينقل الناصح مشاعر الاحترام لشخص المشمول بالرعاية |  |  |  |  |
| 30 | المشمول بالرعاية يشرك الناصح معه فى مشاكله الشخصية |  |  |  |  |
| 31 | يوجه الناصح المشمول بالرعاية للتطوير الشخصي |  |  |  |  |
| 32 | لدى كلا من الناصح والمشمول بالرعاية في كتير من الأحيان تعاملات اجتماعية غير رسمية خارج جلسة العمل |  |  |  |  |
| 33 | يحافظ الناصح على شكوك ومشاعر المشمول بالرعاية معه بسرية تامة |  |  |  |  |
| 34 | يتفاعل الناصح مع المشمول بالرعاية اجتماعيا وخارج العمل |  |  |  |  |
| 35 | يظهر الناصح مهارات الاستماع الجيد في محادثات المشمول بالرعاية |  |  |  |  |
| 36 | يعتبر المشمول بالرعاية الناصح بمثابة صديق له |  |  |  |  |
| 37 | في كثير من الأحيان يتقابلون معا بطريقة غير رسمية بعد العمل |  |  |  |  |
| 38 | المشمول بالرعاية يحترم ويعجب بالناصح |  |  |  |  |
| 39 | يساعد الناصح المشمول بالرعايةو يوجهه لتطوير وفهم نفسه او نفسها |  |  |  |  |

| هل تود إضافة أي شيء حول خبراتك والتي لم نقم بذكرها أعلاه؟ |
| --- |
